# Supplementary material for: Learning curve and surgical outcome of robotic assisted colorectal surgery with ERAS program
Source: Sci Rep. 2022 Nov 29;12:20566. doi: 10.1038/s41598-022-24665-w (PMC9709162; doi:10.1038/s41598-022-24665-w)
Supplement: Supplementary file 2 — Supplementary Information 2. [file 41598_2022_24665_MOESM2_ESM.zip › RAL Raw data-Ñ[▒K/058/OP_ñw╝╨░O▒Kñσ.pdf]

正式摘要(電子簽章：000000209200881)
請選擇...reporter

臺中榮民總醫院

Operation Note

[redacted]

- 手術日期：2021/01/28
- 手術醫師：VS 蔣鋒帆，CR 林陳彥
- Anesthesia : GE
- Position : Lithotomy
- Aseptic agents : Betadine Aqua , Zephnan

Diagnosis

- Pre-operative diagnosis : Malignant tumor of rectum
- Post-operative diagnosis : s/p robotic low anterior resection + loop ileostomy

Surgical Indication

- For removing the primary tumor

Operation

- 1. Robotic (XI) assisted low anterior resection + loop ileostomy
  2. Intraoperative colonoscopy : (-).
  3. High ligation (+) : At IMA root. Left colic artery and IMV were also ligated via robotic endoclips.
  4. Take down from the splenic flexure : (-)
  5. Robotic endoGIA (45-4.3) X2 + EEA (29mm) + OpenGIA (80-4.8) X1 were applied to complete side to end anastomosis
  6. Proximal cut end: 2 cm, distal cut end: > 10 cm
  7. ADHESION BARRIER FILM was placed below the central abdominal wound

Finding

- 1. A 2 x 2 cm, ulcerative tumor at distal rectum. DRE : Finger tip.
  2. No obvious ascites
  3. Mild adhesion was presented between the S-colon, abdominal wall and the nearby small bowel
  4. No obvious lesions were noted over the liver surface
  5. Bilateral doughnuts : intact ; Air leak test: Done and no air leak was found

Procedure

- 1. General endotracheal anesthesia was induced and then the patient was placed in modified Trendelenburg lithotomy position.
  2. Preoperative antibiotics were given. A Foley catheter and nasogastric tube were placed.
  3. The abdomen was prepped and draped in standard sterile fashion.
  4. A transverse incision was made above the umbilicus and a 8 mm trocar was inserted into the peritoneal cavity by open method. Peumoperitoneum was established with CO2 inflation.
  5. Laparoscopy was inserted and then another 8mm trocar was inserted over LUQ area of abdomen. A 12 mm trochar over RLQ area of abdomen. Another 8mm trochar over RLQ of abdomen. Finally, a 5 mm trocar was inserted over RUQ area as an assist-port. The operative findings were mentioned as above.
  6. The sigmoid colon was held by grasper and the peritoneum overlying the mesentery was scored with electrocautery.
  7. The inferior mesentery artery was identified, ligated above the left colic artery with robotic endoclips, and transected.
  8. Dissection was done above the Gerota’s fascia and toward the peritoneal attachments.

- 9. The colon was mobilized from retroperitoneum overlying the sacral promontory and into the pelvis.

  10. The incision was extended laterally and medially across the peritoneal reflection.
  11. The mesorectum was then mobilized using sharp dissection over the level of pelvic floor muscle. Then distal descending colon and rectum were mobilized as possible.
  12. DRE was performed at the same time to make sure the desired resection line is adequate.
  13. The distal rectum site was hold via Vessel loop, then it was divided with Robotic endoGIA (45-4.3) X2.
  14. The umbilicum area wound of abdomen was further extended. We approached into the peritoneal cavity by open method. rectum was pulled out.
  15. The proximal colon was then taken out for carefully inspection to make sure it has an excellent blood supply. The mesenteric fat was gently cleared from the terminal 2-3 mm of the bowel ends.
  16. The field was isolated with moist pad and the bowel opened and sized. It was determined that a 29 mm circular stapler could be accommodated. A small incision was made near the end of the rectum, which then gentally extended to allow the anvil to be inserted. The anvil was penatrated from the lateral side of the rectum. (For later side to end approach)
  17. The anal canal was irrigated with distil water and then the circular stapler was introduced through the anus and pierced through the upper area (The ventral site of the body) of the stapler line.
  18. The proximal bowel with anvil in place (Side) was engaged with the circular stapler (End) and the stapler was then fired. Two complete donuts were obtained and the distal one was submitted to pathology for margin confirmation.
  19. The anastomosis was tested for leaks with irrigation solution and air introduced through the rectum and was found to be patent and intact.
  20. The bleeder was checked thorughly again.
  21. The desired ileum site (About 15~20cm from IC valve) was selected for further ostomy construction.
  22. The 12mm trochar wound over the RLQ area was further extended to creat the site for loop ileostomy.
  23. The ileum was retracted from the desired ostomy site. A window was created under the ileum by dividing the mesentery adjacent to the bowel wall with electrical cautery carefully, with a plastic bridge in placed.
  24. The trochar fascia wounds were all then closed with interrupted sutures via 1-O Vicryl.
  25. ADHESION BARRIER FILM was placed below the central abdominal wound.
  26. The extended fascia wound was closed with interrupted sutures via Num.1 Vicryl.
  27. Massive N/S was applied to irrigate the wound.
  28. The wound was further closed with 3-O Vicryl, and the skin was applied with SurgiSeal, followed by Medipore.
  29. Moist pads were placed and the ileum was opened. Succus and air were aspirated.
  30. The ileostomy was matured with multiple interrupted mucocutaneous suture via 4-0 Monocryl.
  31. The ileostomy was well formed and viable. An ostomy bag was placed
  32. NG tube was removed. The patient tolerated the procedure well and was brought to the POR in stable condition.
- Estimated blood loss: minimum
- 器械數量: 正確
- 紗布數量: 正確

Image

- PAINT
  - abdomen

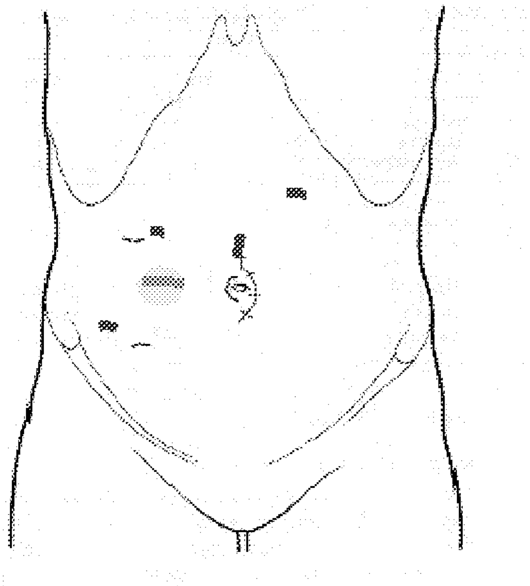

Yellow circle : Loop ileostomy site

- colon & rectum

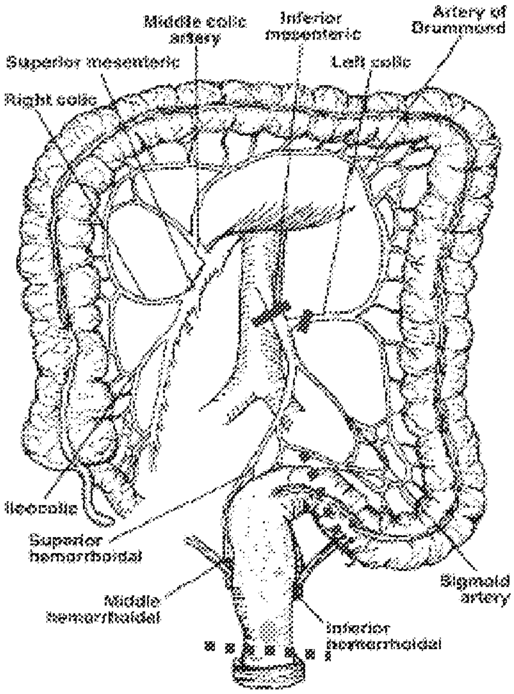

- The resected rectum

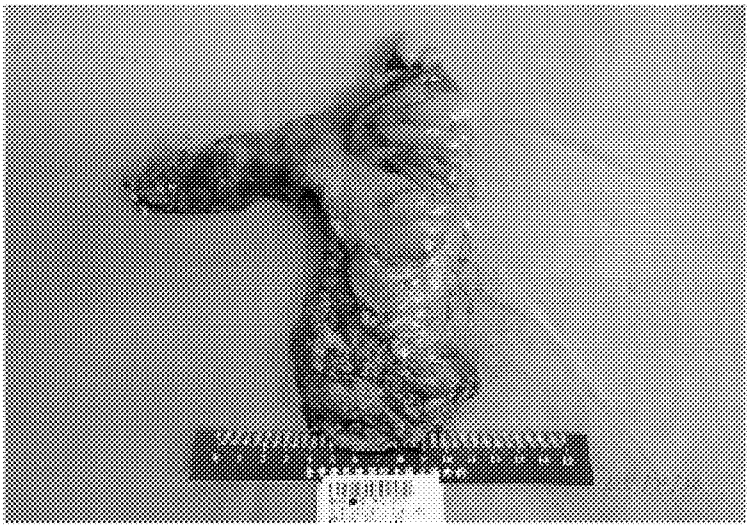

The tumor

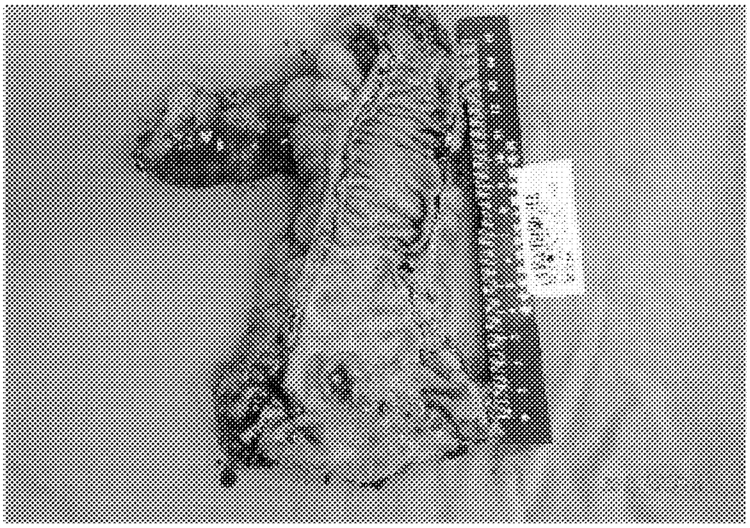

The distal margin was around 2 cm

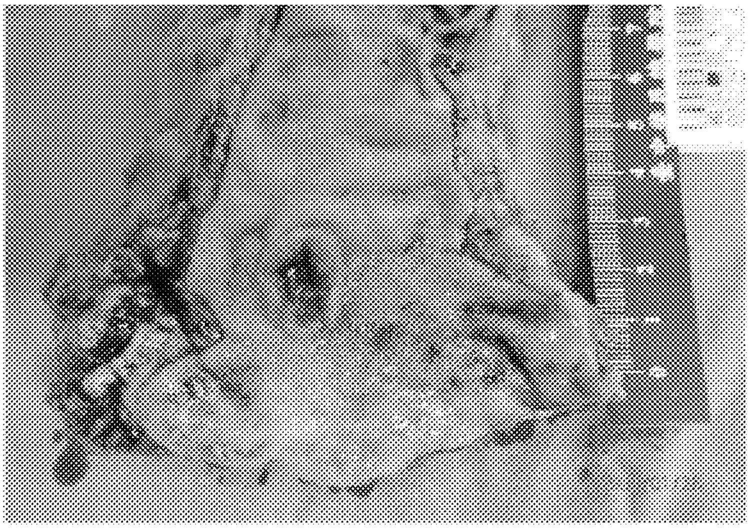

主任 2062F蔣鋒帆 / 醫師 2301H林陳彥

正式摘要(電子簽章：**000000209200881**)

請選擇...reporter

doctorRank:B
